# Reliability of Self-Administered Questionnaire on Dietary Supplement Consumption in Malaysian Adolescents

**DOI:** 10.3390/nu12092853

**Published:** 2020-09-17

**Authors:** Mohamed S. Zulfarina, Razinah Sharif, Ahmad M. Sharkawi, Tg Mohd Ikhwan Tg Abu Bakar Sidik, Sabarul-Afian Mokhtar, Ahmad Nazrun Shuid, Isa Naina-Mohamed

**Affiliations:** 1 Pharmacoepidemiology and Drug Safety Unit, Department of Pharmacology, Faculty of Medicine, Universiti Kebangsaan Malaysia Medical Centre, Jalan Yaacob Latif, Bandar Tun Razak, Cheras 56000, Kuala Lumpur, Malaysia; P94838@siswa.ukm.edu.my (M.S.Z.); shark_811@yahoo.com (A.M.S.); tgikhwancrc@gmail.com (T.M.I.T.A.B.S.); drsam2020@gmail.com (S.-A.M.); anazrun@yahoo.com (A.N.S.); 2 Centre for Healthy Ageing and Wellness, Faculty of Health Sciences, Universiti Kebangsaan Malaysia, Jalan Raja Muda Abd Aziz 50300, Kuala Lumpur, Malaysia; razinah@ukm.edu.my; 3 ProVice-Chancellor Office, Health Campus, Jalan Yaacob Latif, Bandar Tun Razak, Cheras 56000, Kuala Lumpur, Malaysia; 4 Department of Pharmacology, Faculty of Medicine, Universiti Teknologi MARA, Sungai Buloh Campus, Jalan Hospital, Sungai Buloh 47000, Malaysia

**Keywords:** test–retest, nutritional supplement, vitamin, mineral, herbal, natural, botanical, nutraceutical

## Abstract

The repeatability of most questionnaires utilized in previous studies related to the consumption of dietary supplements (DS) among youth has not been well documented. Thus, a simple and easy-to-administer questionnaire to capture the habitual use of DS in the past one year known as the dietary supplement questionnaire (DiSQ) was developed and supported with external reliability evaluation. Analyses were done based on a convenience sample of 46 secondary school students. To elicit information regarding the intake of DS, the questionnaire was partitioned into two domains. The first domain was used to identify vitamin/mineral (VM) supplements, while the second domain was utilized to identify non-vitamin/non-mineral (NVNM) supplements. Cohen’s kappa coefficient (*k*) was used to evaluate the test–retest reliability of the questionnaire. Questionnaire administration to the respondents was done twice whereby a retest was given two weeks after the first test. Between test and retest, the reliability of individual items ranged from moderate to almost perfect for the VM (*k* = 0.53–1.00) and NVNM (*k* = 0.63–1.00) domains. None of the items had “fair” or ”poor” agreement. Various correlation coefficients can be obtained for the DiSQ but are generally reliable over time for assessing information on the consumption of supplements among the adolescent population.

## 1. Introduction

There is no global consensus in terminology on how dietary supplements or supplements in different countries are defined [1,2]. Based on the major regulatory bodies, dietary supplement (DS) is the product or foodstuff that contains dietary ingredients or substances [3] or a concentrated source of nutrients or other substances designed to be taken in small quantities [4,5] either individually or in combination, marketed in the form of pill, tablet, capsule and other similar forms, all of which are intended to supplement the normal diet [3,4,5]. 

The global market of DS is a multibillion-dollar commerce, with the Asia Pacific being the second largest market accounting for 31% of the total market share [6]. Studies established that there are substantial national variations regarding the prevalence of dietary supplement consumption in children and adolescents; 20% in Australia [7], 32% to 37% in the United States [8,9], 35% in Italy [10], 20% to 22% in Japan [11,12] and 33% in South Korea [13]. Specifically, the prevalence of vitamin/mineral supplements (54%) and food supplements (40%) intakes are more pronounced among Malaysian adolescents [14]. Regional differences suggest that the guidelines set by the international and national regulations play one of the key roles in the widespread consumption of supplements [15]. For example, countries of the Association of Southeast Asian Nations (ASEAN) have relatively loose regimes that may lead to easy market access compared to those of other international alliances or regions including the European Union (EU) and Japan [1,2].

Given the widespread and increasing use of DS, the contribution of supplement intake towards one’s health is an important exposure in any health epidemiologic study and overlooking this may lead to an underestimation of the true relationship between the consumption of DS and health condition [16,17,18]. Epidemiological study represents an essential research method in understanding the association between risk factors and disease outcomes with the administration of questionnaires being the most commonly utilized scientific tool for acquiring such information. Indeed, the empirical utility of this approach in epidemiological studies to identify risk factors is apparent [19].

To date, many studies have documented the use of supplements and their associated determinants among adolescents and young adults. Most studies over the past 10 years have been conducted in the United States [8,9], Europe [10,20,21,22] and Australia [7] with limited studies conducted in the Asian countries [12,13,14,23]. However, the external reliability of most questionnaires utilized in earlier studies has not been well documented. To the best of the authors’ knowledge, only one study has demonstrated the test–retest reliability of questionnaires on the use of DS in adolescents [24]. However, the application of dietary methods including Food Frequency Questionnaire (FFQ) used in the previous study required a country-specific nutrient database for DS.

Owing to the scarcity of studies on the reproducibility of questionnaires on the intake of supplements among youth as well as the limitation of local nutrient database, a self-report questionnaire was constructed to offer a simple and easy-to-administer tool provided with an external reliability evaluation. Providing external reliability is essential to ensure that the information provided by the questionnaire is reliable over time. This control process is vital in producing accurate data interpretation and its subsequent use by policy-makers and practitioners to design, implement as well as evaluate policies, practices and intervention strategies in health. Thus, this present study aimed at describing and addressing the degree of consistency of a self-reporting questionnaire on the consumption of DS in a representative group of adolescents aged 15 to 19 years old using test–retest reliability.

## 2. Methods 

A total of 50 national secondary school adolescents (aged 15–19) from the city center of Kuala Lumpur, Malaysia were selected on a convenience basis. The questionnaire was administered to each respondent twice. The elapsed time between the first questionnaire administration and the next was two weeks (14 to 15 days) [25]. The participants took approximately five to fifteen minutes to complete the questionnaire. Oral instruction was also given during each survey, and the respondents were encouraged to seek aid on any items in the questionnaire that they did not fully comprehend. This procedure would allow for the clarification of any ambiguities.

During the re-administration, four participants were not present to complete the second copy of the questionnaire and thus treated as missing data. For this reason, the remaining 46 participants who completed the questionnaire twice were used in the final analysis.

### 2.1. Questionnaire

For ecological validity, the items for dietary supplement questionnaire (DiSQ) were developed and structured on the basis of existing literature and surveys [8,17,18,26]. This step was carefully reviewed by the panel of experts on pharmacology and nutrition. The questionnaire was partitioned into three domains. The first two domains were used to elicit information regarding the intake of DS while the third domain (additional information) was used to inquire about the intake of medicines over the past one year. Specifically, the first domain was used to identify vitamin/mineral (VM) supplements, whereas the second domain was used to identify non-vitamin/non-mineral (NVNM) supplements, which are also known as natural products or nutraceuticals such as herbals/botanicals, amino acids or omega-3 fatty acid.

A closed-ended format was used to collect information on the consumption of supplements and medicines over the past year (yes, no). An open-ended format was introduced for the next question in which the respondents were required to list the names of the products they consumed. For each supplement and medicine listed, the respondents were asked to provide information that included a closed-ended format on frequency of consumption (at least once a day, a few times in a week/more than once a week, once a week, once in a few weeks/one to three times a month), status of consumption or whether or not the subject is still taking the supplement (current, former), and duration of consumption (short-term refers to less than six months, long-term refers to more than six months).

The respondents may incorrectly identify the type of supplement they consume, which suggests that the respondents’ perception of dietary supplements may not match that of the researcher [27]. To amend this, a card that listed the examples of VM supplements for the first domain and NVNM supplements or natural products for the second domain was introduced (Appendix A). 

### 2.2. Sample Size

The minimum sample size required was determined using the tabulated contingency table by [28], which was calculated based on the formula by [29]. The minimum sample size required was derived based on the pre-specified power of 80%, alpha of 0.05 two-sided and the expected Cohen’s kappa (*k*) coefficient (*k*_2_ is 0.42) for every item when no agreement was assumed for the test–retest at the first place (*k*_1_ is 0.0). Thus, a minimum sample of 43 respondents was required based on the assumption that the proportions in each category were proportionate to one another.

### 2.3. Ethics

This initial work is a part of the larger population-based study where the details of the methodology have been published previously [30]. The participants involved in this reliability study were not included in the analysis mentioned above. Formal approval was obtained from the Universiti Kebangsaan Malaysia Medical Centre (UKMMC) research committee (UKM 1.5.3.5/244), Ministry of Education (MOE), the Department of State Education (JPN), and school authorities. Participation in this study was completely voluntary and parents/guardians who preferred their children not to participate in the study were required to sign and return the negative consent form.

### 2.4. Statistics

All statistical analyses were conducted using the Stata Statistical software version 15 (Stata Corporation LP, College Station, TX, USA). The percentage of agreement and Cohen’s kappa (*k*) coefficient were assessed for each item and its sub-components. Percent agreement was calculated by dividing the number of agreement scores with the total number of scores. Unweighted *k* statistic was used to evaluate nominal variables, whereas weighted *k* statistic with quadratic weighting was used to evaluate ordinal variables. Four respondents with missing data were excluded from all computations.

The strength of agreement was interpreted with due caution according to the reference values described by Landis and Koch in which Cohen’s *k* coefficient of less than 0.20 was considered as poor agreement, 0.21–0.40 as fair agreement, 0.41–0.60 as moderate agreement, 0.61–0.80 as substantial agreement, and 0.81–1.00 as almost perfect agreement [31]. 

## 3. Results

From the initial sample of 50 respondents, only 46 (92%) individuals completed the questionnaire twice. Among the 46 respondents, 50% were male students and 50% were female students (mean age 16.9 ± 0.8). The majority of the volunteers were of Malay race (85%). The characteristics of adolescents who completed the survey are shown in Table 1. Table 2 shows the prevalence of DS users in the first questionnaire administration and the follow-up after two weeks. The prevalence of the VM supplement use was 39% for the baseline administration and 37% for the re-administration. On both occasions, vitamin C was the most popular subtype of the VM supplements among the adolescents (baseline: 72%; follow-up: 71%). Regarding the NVNM supplement intake, the prevalence for the baseline administration was 46% with 48% for the re-administration, while the most consumed nutraceuticals on both occasions were herbs and botanicals (baseline: 62%; follow-up: 50%). Additionally, the second most consumed nutraceutical products in the baseline administration and re-administration, respectively, were honey and bee products (baseline: 48%; follow-up: 36%).

The reliability coefficient for the VM supplement use is presented in Table 3. The result of the main item, which was the first listed item in the table, displayed an ”almost perfect” test–retest reliability (*k* = 0.95). For the sub-item, the item on the supplement’s name presented the highest agreement (*k* = 0.68–1.00), while the item on the duration of consumption showed the lowest agreement (*k* = 0.53–1.00) between test and retest. None of the items had kappa values of less than 0.40 (“fair” or ”poor” agreement). The percent agreement for the VM supplement use ranged from 87.0% to 100.0%.

Table 4 shows the reliability coefficient for the NVNM supplement consumption. The kappa value of the main item (*k* = 0.78) denoted a “substantial” test–retest reliability. For the sub-items, the item on the frequency of intake presented the highest agreement (*k* = 0.79–0.97), while the item on the status of consumption presented the lowest agreement (*k* = 0.63–0.88) between the first and second surveys. All items had kappa values of more than 0.60 (indicating a “substantial” to an “almost perfect” agreement). The percent agreement for the NVNM supplement use ranged from 77.3% to 100.0%.

Table 5 shows the reliability coefficient for the medicine intake. The kappa statistics of the main item indicated an “almost perfect” test–retest reliability (*k* = 0.93). For the sub-item, the items listed on the name of medicine and status of consumption presented the highest agreement (*k* = 0.48–0.94), while the item on the duration of consumption presented the lowest agreement (*k* = 0.22–0.69) between test and retest. All items had kappa values of more than 0.40 (indicating a “moderate” to an “almost perfect” agreement) except for the item on the duration of consumption. The percent agreement for the medicine intake ranged from 89.1% to 100%.

## 4. Discussion

Across the three domains, the agreement values of *k* for the two-week interval indicated that all individual items have moderate to almost perfect repeatability. This lies in the preferable measure of agreement except for the item on the duration of the medicine consumption. Thus, it can be deduced that the test–retest reliability of the current self-reported supplement consumption is varied but generally reliable over time.

It was difficult to compare the current results directly with those of previous studies due to the variations in the context of questions and type of response alternatives used in the questionnaire. However, the repeatability of responses was in part comparable to that obtained in another study [24]. The percent agreement for the overall dietary supplement use was 91.7% with *k* = 0.62 for the reported category, while for the corrected category, the percent agreement was 89.8% with *k* = 0.57. 

A report suggested that a brief questionnaire can precisely capture the data on the use of the frequently consumed supplements but may not perform well for the less frequently consumed supplements [32]. On the contrary, this present study demonstrated that the reliability estimate for the NVNM supplement consumption was equally comparable to the VM supplement consumption. Concerning the main item (the first question asked in the questionnaire), the reliability estimate regarding the NVNM supplement consumption was lower than that of the VM supplement consumption and medicine use. This result could be partly supported by the fact that supplements are more relevant to the older generation than to the younger generation (prevalence rate) [33]. The prevalence of supplement users may have an influence (albeit small) on the percent agreement estimate between the two questionnaires. The *k* value would probably be higher in a population with a high prevalence of users. Moreover, unlike the VM products, most NVNM products are consumed intermittently (behavioral differences). Thus, adolescents may face difficulty in recalling the supplements they consumed infrequently.

As shown in this study, the values of Cohen’s *k* statistic for the sub-items 2 to 5 were lower compared to that of the first item under the same domain. It is interesting to note that most teenagers took the supplements as requested by their parents [14]. It was assumed that this reason might also be applied to the NVNM supplement consumption. Although the underlying causes were unclear, adolescents may be aware but did not pay attention to what supplements were given to them since it was not by their own free will or self-awareness [23]. As a result, they were unable to recall specific details such as the type of supplement they consumed because recalling an involuntary action precisely may require more complex cognitive demands [34].

The questions on medicine use were also included as additional information for which the low agreement between test and retest was in line with the previous findings concerning adult respondents [26]. This study postulated that the agreement between test and retest would likely reflect the dependence on time and situation during the period covered [35]. In most cases, medicines are consumed at an interval or when pain is present [26]. This may imply that the agreement between the two responses resulted from the measure of the consistency of the medication use rather than the measure of reliability of the instrument [32].

The open-ended format was chosen for the item on the supplement’s name as it allowed for a wide coverage of various types of DS consumed to be recorded. An example of DS includes the *Habbatus sauda*, which is commonly known as black cumin that is native to Southwest Asia. Another example is long jack, locally known as Tongkat Ali, which is indigenous to Southeast Asia and culturally used as folk medicine. By having this format, diverse types of supplement can be documented. Moreover, the DiSQ was developed to cover a supplement intake for one year, and consequently, the previous supplement intake could be missed. Nevertheless, it can still be highlighted that the DiSQ is able to capture the current habitual information on both daily/regular and intermittent/occasional supplement users.

There were some limitations discovered based on the findings of this study. First, the sampling was based on a convenience sample. Second, the questionnaire lacked the questions on an actual dosage level. This was made on account of the insignificant actual dosage level considered in many epidemiologic studies as long as the intakes between two instruments were similar [32]. In addition, the determination of total intake, which includes nutrients consumed from DS, requires a country-specific nutrient database; thus, the inquiry on dosage level was not considered in this study. On the contrary, countries with food database integrated with nutrients from DS have the advantage in measuring total intake that covers nutrients from DS.

This work represents an initial step in a larger-scale study to determine the lifestyle and health outcomes among adolescents specifically or the younger generation in general. Consequently, this current study will serve as a baseline test from which the application to other age groups or populations of interest (adult or elderly) is possible after establishing the test–retest reliability. It is recommended that in the subsequent results evaluation, the items with “fair” kappa statistic should be considered with due caution, modified, or possibly removed from the questionnaire. Further research that appraises additional validity and reliability of the questionnaire is warranted.

## 5. Conclusions

In accordance with the reference criteria adopted, this study has demonstrated that the concordance or correlation coefficient values of the questionnaire are varied but generally satisfactory and reliable over time. This study will serve as a starter for other new studies related to this topic and DiSQ can be a valuable tool in future studies that aim to investigate the association between various risk factors such as supplement consumption, demographic characteristics, and lifestyle particularly in the adolescent population.

## Figures and Tables

**Table 1:** Characteristics of study participants.

	Mean ± SD/*n* (%)
All	46
Age	16.9 ± 0.8
Gender	
Male	23 (50.0)
Female	23 (50.0)
Ethnicity	
Malay	39 (84.8)
Non-Malay *	7 (15.2)

* Other races including Chinese and Indian were grouped under one category due to low representation.

**Table 2:** Descriptive data on the baseline administration and re-administration.

	Baseline Prevalence, *n* (%)	Follow-Up Prevalence, *n* (%)
Vitamin/mineral		
Number of users	18 (39.1)	17 (37.0)
Number of supplements		
1 type	13 (72.2)	13 (76.5)
2 types	5 (27.8)	4 (23.5)
>3 types	0 (0.0)	0 (0.0)
Type of DS		
Single vitamin	14 (77.8)	13 (76.5)
Single mineral	3 (16.7)	5 (29.4)
Combination of vitamin(s) and mineral(s) ^*^	7 (38.9)	4 (23.5)
Non-vitamin/non-mineral		
Number of users	21 (45.7)	22 (47.8)
Number of supplements		
1 type	8 (38.1)	10 (45.5)
2 types	9 (42.9)	8 (36.4)
>3 types	4 (19.1)	4 (18.2)
Type of DS		
Honey and bee products	10 (47.6)	8 (36.4)
Fish oil/omega-3	7 (33.3)	7 (31.8)
Meat essence	5 (23.8)	5 (22.7)
Herbs and botanicals	13 (61.9)	11 (50.0)
Protein formula	2 (9.5)	2 (9.1)
Others ^‡^	2 (9.5)	3 (13.6)
Unspecified ^#^	-	1 (4.5)

DS = dietary supplement * combined preparations containing vitamin and mineral such as multivitamin, multi-mineral, multivitamin multi-mineral, or any type of combination ^‡^ including functional food ^#^ treated as missing data.

**Table 3:** Cohen’s kappa (*k*) coefficient for the vitamin/mineral (VM) supplement consumption.

Items	Response Alternatives	*n*	Percentage Agreement (%)	Kappa Coefficient
Used or taken any vitamins/minerals in the past one year	Yes/no	46	97.8	0.95
Name of supplement	*	46	93.5–100.0	0.68–1.00
Still take	Yes/no	46	91.3–100.0	0.67–1.00
Frequency of intake	At least once a day/more than once a week/once a week/one to three times a month	46	87.0–97.8	0.64–0.80
Took continuously for more than 6 months	Yes/no	46	87.0–100.0	0.53–1.00

* Open-ended answer.

**Table 4:** Cohen’s kappa (*k*) coefficient for the non-vitamin/non-mineral (NVNM) supplement consumption.

Items	Response Alternatives	*n*	Percentage Agreement (%)	Kappa Coefficient
Used or taken any NVNM in the past one year	Yes/no	46	89.1	0.78
Name of supplement	*	45	84.4–97.8	0.77–0.87
Still take	Yes/no	43	79.1–97.8	0.63–0.88
Frequency of intake	At least once a day/more than once a week/once a week/one to three times a month	46	77.3–95.7	0.79–0.97
Took continuously for more than 6 months	Yes/no	46	81.8–100.0	0.69–1.00

* Open-ended answer.

**Table 5:** Cohen’s kappa (*k*) coefficient for medicine consumption.

Items	Response	*n*	Percentage Agreement (%)	Kappa Coefficient
Used or taken any medicines in the past one year	Yes/no	46	97.8	0.93
Name of medicine	*	46	95.7–100.0	0.48–0.94
Still take	Yes/no	43	95.7–100.0	0.48–0.94
Frequency of intake	At least once a day/more than once a week/once a week/one to three times a month	46	91.1–100.0	0.42–0.82
Took continuously for more than 6 months?	Yes/no	46	89.1–100.0	0.22–0.69

* Open-ended answer.

## Appendix A

**Table A1:** Examples of VM and NVNM supplements.

Vitamin/Mineral	Non-Vitamin/Non-Mineral
Vitamin C	Fish oil/omega-3/omega-6
B vitamins or B complex	Spirulina
Folic acid/vitamin B6	Evening Primrose Oil
Calcium	Honey/bee products
Vitamin D	Co-enzyme Q10
Zinc	Chicken/Fish essence
Iron	Sea Cucumber
Multivitamin and/or Multimineral	Fiber
Vitamin A	Protein/amino-acids
